# Community supervision during Oregon’s partial decriminalization Measure 110: Criminal legal system involvement, overdose, and naloxone access

**DOI:** 10.1016/j.dadr.2026.100430

**Published:** 2026-03-15

**Authors:** Hope M. Smiley-McDonald, Esther O. Chung, Lynn D. Wenger, Danielle Good, Gillian Leichtling, Barrot H. Lambdin, Alex H. Kral

**Affiliations:** a RTI International, Research Triangle Park, NC, United States; b RTI International, San Francisco, CA, United States; c Comagine Health, Portland, OR, United States

**Keywords:** Community corrections, Parole/probation, Drug decriminalization, Oregon Measure 110, People who use drugs

## Abstract

**Background:**

In 2020, the U.S. state of Oregon passed Measure 110 (M110), which aimed to address substance use disorder as a public health issue and reduce disparities in the criminal legal system by decriminalizing personal drug possession and increasing services. The impact of partial drug decriminalization on individuals under community supervision—whose release conditions often prohibit drug use and who M110 excluded—is understudied.

**Methods:**

We used targeted sampling to recruit and survey people who use drugs (PWUD; N = 468) in eight Oregon counties in 2023. We compared PWUD under community supervision to those who were not to assess opioid-related overdose, naloxone access, and law enforcement engagement.

**Results:**

Compared to PWUD who were not under community supervision, those under supervision had higher prevalence of past year opioid-related overdose. There were no differences by naloxone access. Eighty-two percent (82%) of PWUD on community supervision were stopped by law enforcement in the past year. PWUD on community supervision were more likely than those not on community supervision to report in the past year being searched by law enforcement at least once (adjusted prevalence differences [APD]=0.33; 95% CI: 0.23, 0.43), spent time in jail at least once (APD=0.33; 95% CI: 0.23, 0.43), and to have concerns about getting into trouble if they called 911 for a drug-related health issue (APD=0.12; 95% CI: 0.00, 0.18).

**Conclusion:**

Under M110, Oregon PWUD under community supervision experienced more police engagement and overdoses. Findings have implications for less police presence at overdose scenes, greater access to naloxone and support services, and protections under future decriminalization laws.

## Highlights

• Community-supervised PWUD had a high prevalence of past year overdose.
• The majority of community-supervised PWUD (82%) reported high levels of past year police engagement.
• Community-supervised PWUD had concerns about being sanctioned during drug decriminalization.
• Community-supervised PWUD feared legal trouble regarding calling 911 for an overdose.

## 1 Introduction

Drug use and overdose remain significant global public health and safety issues, with an estimated 64 million people worldwide suffering from drug use disorders in 2023 (UNODC, 2025) and 0.6 million deaths attributed to drug use in 2024 (World Health Organization, 2024). The United Nations reports that drug use or possession is a criminal offense in 40 % of the 94 countries with available data (United Nations, 2026). In 2023, an estimated 6.1 million people worldwide had police contact (e.g., suspected, arrested, or cautioned) for drug-related offenses (UNODC, 2025).

The U.S. has high rates of drug-related police contact, incarceration, and community supervision. In 2024, the U.S. Federal Bureau of Investigation reported that about 1.6 million arrests were linked to drug offenses (Federal Bureau of Investigation, 2026). There were an estimated 5.4 million persons under supervision in adult correctional systems across the U.S. in 2022; of these, about 3.7 million were under community supervision via probation or parole (Buehler, Kluckow, 2024). An estimated 58% of state prisoners and 63% of sentenced jail inmates meet the criteria for drug dependence (Bronson et al., 2017). Carceral experiences that include being on community supervision have significant negative consequences for employment, education, housing, public benefits, health, mental health, and future overdose risk in the U.S., England, and Canada (Brinkley-Rubinstein et al., 2019, Brooker et al., 2020, Bryson et al., 2019, Bryson et al., 2021, Cohen et al., 2022, Fearn et al., 2016, Grebely et al., 2017, Knittel et al., 2019, Lewis and Ford, 2024, Lorvick et al., 2018, Menza et al., 2025, Morgenstern et al., 2008, Nino et al., 2023, Sirdifield et al., 2020, Vaughn et al., 2012, Winkelman et al., 2020, Zhang et al., 2022).

Numerous studies show an association between criminal legal system (CLS) involvement and overdose. Binswanger et al. (2007) found that people are over 120 times more likely to overdose within the first 2 weeks after being released from incarceration, a finding echoed elsewhere (Brinkley-Rubinstein et al., 2019, Hartung et al., 2023, Hoover et al., 2023). Other studies show community supervised populations have high prevalence of overdoses more generally (Boulger et al., 2022, Cropsey et al., 2013, Wakeman et al., 2009). People who use drugs (PWUD) are reluctant to call 911 during overdoses because they fear arrest (Baker et al., 2024, Saloner et al., 2025) or violating parole or probation conditions (Saloner et al., 2025). PWUD are also concerned about carrying and using naloxone to reverse overdoses for fear of legal repercussions (Byles et al., 2024). Other research shows that police arrest and incarcerate people after overdose incidents in the U.S. and Canada, despite Good Samaritan Laws (GSLs) (Dickson-Gomez et al., 2025, Richardson et al., 2023, Thompson et al., 2024, Wettemann et al., 2025).

In November 2020, Oregon passed Ballot Measure 110 (M110), a pioneering partial drug decriminalization policy that shifted substance use disorder from a public safety issue to public health concern. By prioritizing services and recovery over incarceration, M110 sought to reduce overcrowding in the CLS and address longstanding racial disparities in drug enforcement (Drug Addiction Treatment and Recovery Act, 2020). Oregon is the first and only U.S. state to partially decriminalize possession of personal amounts of all drugs. Prior to M110, there were about 16,800 arrests for drug possession in Oregon annually (Henderson et al., 2023). National statistics show that Oregon’s incarceration (2390 vs. 2400 per 100,000 U.S. adult residents) (Minton et al., 2021) and community supervision (1767 vs. 1701 per 100,000 U.S. adult residents) (Oudekerk and Kaeble, 2021) rates were comparable to the U.S. in 2019 (Minton et al., 2021).

Though M110’s partial drug decriminalization component was implemented in February 2021, the funding for substance use treatment, harm reduction, and housing services was not disbursed to Oregon’s 36 counties via M110’s established Behavioral Health Resource Networks until August 2022. Consequently, there was an 18-month delay between partial drug decriminalization policy implementation and treatment and support resource allocations (Good et al., 2023). Early assessments of M110’s impact on Oregon’s CLS revealed a decline in arrests for possession of controlled substances following implementation (Davis et al., 2023, Russoniello et al., 2023). Analyses of arrest and crime data from 2017 to 2023 indicated that police stops, searches, and possession arrests were decreasing before COVID-19, starting in 2017 when certain drugs were defelonized; that trend persisted through COVID-19, which coincided with M110’s rollout (Henderson et al., 2023, Henderson et al., 2024). During Oregon’s partial drug decriminalization, survey data showed that PWUD in Oregon experienced high levels of police engagement, including stops and arrests (Smiley-McDonald et al., 2024), while qualitative data revealed PWUD perceived high levels of unpredictability and backlash among law enforcement in drug-related policing, reinforcing PWUDs’ mistrust of law enforcement and creating barriers to police-mediated services (Good et al., 2025). Oregon’s law enforcement leadership and officers perceived that M110 increased crime and diminished their ability to build and investigate cases (Henderson et al., 2023, Smiley-McDonald et al., 2023).

Since M110’s policy language excluded individuals under community supervision, M110’s partial drug decriminalization did not apply to those on probation or parole whose release terms mandated drug abstinence. Qualitative interviews with Oregon law enforcement revealed police coercing this “legacy” population to provide information about drug sellers in exchange for leniency on their probation/parole violations (Smiley-McDonald et al., 2023). Because Oregon is the sole U.S. state to adopt a drug decriminalization policy of M110’s type and scale, there is limited understanding regarding the effects of such legislation on populations under community supervision. This knowledge gap persists even in nations with more established decriminalization frameworks, likely due in part to varying carceral systems and police practices. Portugal’s drug decriminalization law had little to no effect on the country’s “suspended prison” (i.e., probation) population since arrests for possession were uncommon before the 2001 law was established (Laqueur, 2015). Recent research has examined police engagement with PWUD in the context of British Columbia’s 3-year partial drug decriminalization pilot in Canada (Kelsall et al., 2026) and Denmark’s 2004 decriminalization policy involving warnings for socio-economically disadvantaged PWUD (Houborg et al., 2026). Yet missing from recent studies is any direct or indirect focus on community supervised populations in the context of drug decriminalization laws.

This analysis uses a street-based sample of Oregonians who used drugs in 2023 to assess opioid-related overdose, naloxone access, law enforcement engagement, and experiences among individuals who were under community supervision during Oregon’s partial drug decriminalization period, which lasted from February 1, 2021, through August 31, 2024. This study fills research gaps by examining the challenges faced by those under community supervision under a partial drug decriminalization law that excluded them and considers the implications for overdose risk and CLS involvement.

## 2 Methods

### 2.1 Data collection procedures

The findings reported herein are part of a large mixed-methods study evaluating Oregon’s M110. The data were collected using a cross-sectional study design in which respondents completed a one-time quantitative survey, conducted in-person by trained interviewers who read questions aloud and entered answers directly into a tablet with a computer-assisted personal interviewing program (Blaise, Statistics Netherlands, The Hague, Netherlands). The research team collaborated with partner agencies that assist PWUD or directly contacted individuals at homeless encampments and other common sites (e.g., community recycling centers) to enlist participants. Surveys were conducted in private outdoor settings such as parks or encampments, and indoors at partner agencies when available. Every location ensured accessibility and a welcoming environment. The surveys took between 20 and 40 min to complete, and included questions about participant demographics, substance use, opioid-related overdose experiences, CLS involvement, and M110 knowledge. RTI International’s Institutional Review Board approved all study procedures.

### 2.2 Sample

A community-based, cross-sectional survey of people who use drugs was conducted between March and November 2023 across eight of Oregon’s 36 counties. These counties were selected for their population density and geographic diversity and included six non-urban counties (i.e., Coos, Douglas, Jackson, Josephine, Umatilla, and Union) and two urban counties (i.e., Lane and Multnomah). Participants were eligible for the study if they were (1) 18 years or older and (2) had injected or smoked heroin, fentanyl, methamphetamine, or cocaine in the past 30 days. All participants provided informed consent prior to data collection and remunerated $20 for participation.

**Fig. 1:**
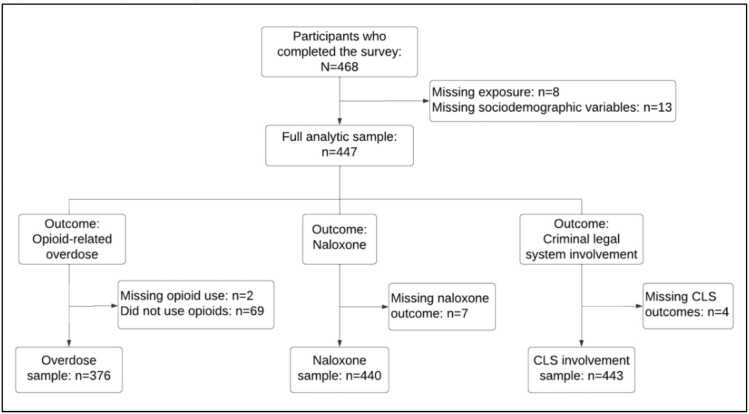
**Analytic Sample Flowchart, Notes:** For overdose outcomes, the analytic sample was restricted to those who ever used opioids. For naloxone and CLS involvement outcomes, there were no restrictions.

There were 468 PWUD who completed the survey. For this study, we had three analytic samples based on our outcomes of interest: opioid-related overdose, naloxone access, and CLS involvement (Fig. 1). Eight participants were missing data on community supervision and an additional 13 had missing data on sociodemographic variables. For opioid-related overdose outcomes, we restricted our analytic sample to those who ever used opioids (n = 376), with 2 participants with missing data on opioid use. For naloxone outcomes, we made no restrictions because naloxone is a lifesaving intervention for opioid and non-opioid users (n = 440), and 7 participants were missing naloxone data. We included all participants for CLS involvement (n = 443); four had missing CLS data.

### 2.3 Measures

#### 2.3.1 Community supervision

Participants were asked whether they were on any of the following types of community supervision in the last 12 months: probation, post-prison supervision/parole, supervised release, or other community supervision. We defined any community supervision in the past year (yes/no) if participants responded affirmatively to any of the options.

#### 2.3.2 Outcomes

Our primary outcomes of interest were opioid-related overdose and naloxone access. Participants were asked “Have you ever had an opioid-related overdose? By overdose we mean a time when you lost consciousness, and someone had to do something to bring you back.” If participants responded “Yes,” they were asked whether they experienced an opioid-related overdose in the past year. We created a binary variable for any opioid-related overdose in the past year and categorized those who had never experienced an opioid-related overdose as “No.”

We defined past year naloxone access using two measures: receiving naloxone and being able to easily access their naloxone. For receiving naloxone, participants were asked “Have you ever gotten naloxone to carry with you (including injectable, intranasal Narcan, and/or autoinjector of naloxone) for use on yourself or others?” If participants responded “Yes,” they were asked whether they received naloxone in the past year. We created a binary variable for receiving naloxone in the past year (yes/no) and categorized those who had never received naloxone as “No.” For accessible naloxone, participants were asked “Could you access your naloxone within a couple of minutes if you needed to use it to reverse an overdose?” We created a binary variable for whether their naloxone was easily accessible (yes/no) and categorized those who did not receive naloxone as “No”.

Our secondary outcomes of interest were experiences with CLS involvement. Participants self-reported the number of times in the past year that (1) law enforcement stopped or questioned them, (2) law enforcement searched them, their car, or their stuff, and (3) they were sent to jail. For each outcome, we created binary variables to indicate if participants experienced the outcome at least once in the past year. Participants were asked how concerned they were about getting in trouble with the law if they called 911 for an overdose or other drug-related health issue. Response options included: (1) Not concerned at all; (2) Not very concerned; (3) Neutral; (4) Somewhat concerned; (5) Extremely concerned. Most participants selected either “Not concerned at all” or “Extremely concerned” and there was limited heterogeneity in other responses. Therefore, we created a binary variable for whether participants were concerned about calling 911 for an overdose (yes/no) by grouping “Somewhat concerned” and “Extremely concerned” as “Yes” and “Not concerned at all”, “Not very concerned’ and “Neutral” as “No”.

#### 2.3.3 Covariates

We selected the following sociodemographic covariates for all models *a priori*: gender, race-ethnicity, housing status, and county of interview. Participants self-reported their gender, which was categorized into cisgender women, cisgender men, and gender expansive (transgender, non-binary, Two-Spirit, agender/no gender, and other). Participants were asked to select their race-ethnicity with the following options: White, Black/African American, Asian, Native Hawaiian/Pacific Islander, Hispanic or Latinx, Native American/Alaska Native, Middle Eastern/North African, or Other. We classified participants as experiencing unstable housing if they reported experiencing at least one of the following: (1) “In the last 12 months, have you spent one or more nights sleeping outdoors on the streets, in a park, in a vehicle or in a tent?” and (2) “In the last 12 months, have you spent one or more nights in a shelter, couch surfing, or staying in a temporary hotel or motel?” The response categories for these questions included: Yes, No, Don’t Know, and Refused. The county in which participants were interviewed was categorized into Urban (Multnomah and Lane counties) and Non-urban (Coos, Douglas, Jackson, Josephine, Umatilla, and Union). We adjusted for other covariates depending on the outcome model. Additional covariates included years of opioid use, participant age, and whether participants experienced an opioid-related overdose in the past year. Years of opioid use was operationalized by subtracting the youngest age at which participants reported using fentanyl, heroin, or opioid pills from their current age. Participant age was categorized into the following groups: 18–34, 35–44, and 45 or older.

### 2.4 Statistical analysis

Descriptive statistics are presented in medians and standard deviations for continuous variables and frequencies and percentages for categorical outcomes. We conducted multivariable models to estimate associations between any community supervision and outcomes. For binary outcomes, we estimated adjusted prevalence differences (aPD) and 95% confidence intervals (CI) using linear-binomial models. For count outcomes, we estimated predicted differences and 95% CI using negative binomial models. We selected confounders *a priori* to include in our multivariable analyses based on each outcome. All models adjusted for gender, race-ethnicity, housing status, and county of interview. For the opioid-related overdose outcome, we restricted our analytic sample to participants that had ever used opioids (n = 376) and adjusted for years using opioids instead of age given overdose risk is more directly related to drug use history than chronological age. We made no sample restrictions for naloxone and CLS models. For naloxone outcomes, we adjusted for age and whether participants experienced an opioid-related overdose in the past year since individuals with recent overdoses may be more likely to seek and be offered naloxone. For CLS outcomes, we adjusted for age and removed housing status from the model due to collinearity. Given sample size restrictions, we were unable to assess gender and race-ethnicity disparities. We compared White participants to all other participants, and we grouped cisgender women and gender expansive participants and compared to cisgender men. Statistical analyses were conducted in Stata, Version 18 and graphics were created in R (R Core Team, 2021, StataCorp, 2023).

## 3 Results

Demographic data by community supervision status in the past year is presented in Table 1. Among Multiracial participants, American Indian/Alaska Native and White was the most common (n = 29, 46%), followed by Hispanic/Latinx and White (n = 9, 14%) and Black and White (n = 5, 8%) (data not shown).

**Table 1:** Sample characteristics of people who use drugs in Oregon by community supervision in past year, (N = 443).

**Characteristic**	**Any Past Year Community Supervision**		**Total** **N = 443**	**P-value**
	***No*** ***327 (73.8%)***	***Yes*** ***116 (26.2%)***		
Age (median [IQR])	40 (31−52)	40 (31−49)	40 (31−52)	0.518
Age categories				
18–34	114 (72.2%)	44 (27.8%)	158 (35.7%)	0.833
35–44	87 (74.4%)	30 (25.6%)	117 (26.4%)	
45–54	126 (75.0%)	42 (25.0%)	168 (37.9%)	
Gender				
Cisgender women and gender expansive*	116 (78.4%)	32 (21.6%)	161 (36.3%)	0.040
Cisgender man	199 (70.6%)	83 (29.4%)	282 (63.7%)	
Racial groups				
White	215 (72.6%)	81 (27.4%)	296 (66.8%)	0.832
Black	23 (74.2%)	8 (25.8%)	31 (7.0%)	
Asian	1 (100.0%)	0 (0.0%)	1 (0.2%)	
Native Hawaiian/Pacific Islander	4 (80.0%)	1 (20.0%)	5 (1.1%)	
Latinx/Hispanic	17 (73.9%)	6 (26.1%)	23 (5.2%)	
Native American/Alaska Native	16 (84.2%)	3 (15.8%)	19 (4.3%)	
Multiracial**	46 (73.0%)	17 (27.0%)	63 (14.2%)	
Other	5 (100.0%)	0 (0.0%)	5 (1.1%)	
White vs. non-White				
White	215 (72.6%)	81 (27.4%)	296 (66.8%)	0.423
Non-White or Multiracial	112 (76.2%)	35 (23.8%)	147 (33.2%)	
Unstable housing in past year				
No	21 (84.0%)	4 (16.0%)	25 (5.6%)	0.233
Yes	306 (73.2%)	112 (26.8%)	418 (94.4%)	
Years using opioids (median [IQR]) (n = 376)	15 (9−24)	16 (9−25)	15 (9−24)	0.859
County of interview				
Urban	201 (75.3%)	66 (24.7%)	267 (60.3%)	0.387
Non-urban	126 (71.6%)	50 (28.4%)	176 (39.7%)	

Table Notes: P-values were from a pooled *t*-test for continuous variables and a Pearson chi-square test for categorical variables. IQR=Interquartile range.

* There were 13 participants that identified as gender expansive (non-binary, transgender, Two-Spirit) and only 1 reported being under community supervision.

** Among Multiracial participants, American Indian/Alaskan Native and White was the most common (n = 29, 46%), followed by Hispanic/Latinx and White (n = 9, 14%) and Black and White (n = 5, 8%).

### 3.1 Experiences of community supervision

One quarter of PWUD (n = 116; 26%) in our sample reported past year community supervision. Across 116 participants reporting that they were on some type of community supervision, 60 were on probation (52%), 33 were on post-prison supervision/parole (29%), 13 were on some other type of supervised release (11%), and 10 were on more than one type of supervised release (9%). Nearly two-thirds (63%) reported they would get sanctioned if their probation officer found out they were using or in possession of drugs, and 66% indicated that they would be comfortable asking their probation/parole officer for a referral to substance use disorder treatment. Forty-three percent of participants on community supervision indicated that providing urine samples was a requirement of their release. Additionally, 40% reported that they would face sanctions if their community supervision officer discovered they were frequenting areas associated with drug use, and 39% stated they would face sanctions if their community supervision officer learned they were socializing with PWUD.

### 3.2 Opioid-related overdose and naloxone access

**Fig. 2:**
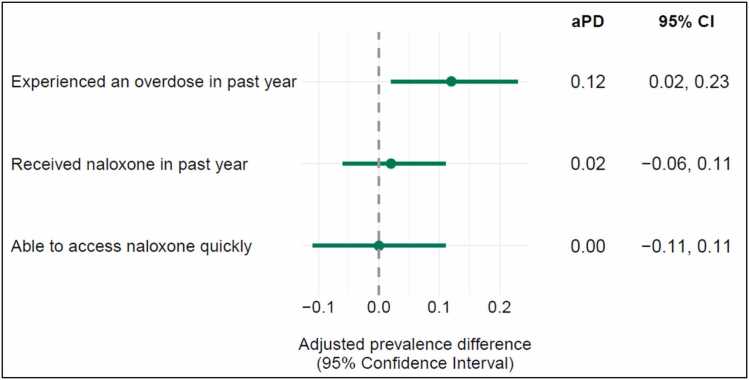
**Associations between any community supervision and opioid-related overdose and naloxone access among people who use drugs in Oregon,** Notes: All models controlled for gender, race, housing status, and county of interview. The overdose model was restricted to participants that had ever used opioids and additionally adjusted for years of opioid use. The naloxone models were not restricted and additionally adjusted for age and whether participants overdosed in the past year. Abbreviations: adjusted Prevalence difference (aPD); Confidence interval (CI).

One-third (34%) of PWUD under community supervision reported experiencing a past year opioid-related overdose (Table 2). Compared to PWUD who were not under community supervision in the past year, those who were on supervision had a 12-percentage point higher prevalence of a past year opioid-related overdose (Fig. 2; 95% CI: 0.02, 0.23). There were no differences in receiving naloxone to carry by community supervision (0.02 (95% CI: −0.06, 0.11) or in ease of access to naloxone (0.00 (95% CI: −0.10, 0.11)).

**Table 2:** Bivariate relationships between community supervision and outcomes among people who use drugs in Oregon.

Outcome	Any past year community supervision		Total	p-value
	**No**	**Yes**		
Overdose outcome	*277 (73.7%)*	*99 (26.3%)*	*N = 376*	
Experienced an overdose in past year				
No	213 (76.9%)	65 (65.7%)	278 (73.9%)	0.029
Yes	64 (23.1%)	34 (34.3%)	98 (26.1%)	
Naloxone outcomes	*325 (73.9%)*	*115 (26.1%)*	*N = 440*	
Received naloxone in past year				
No	76 (23.4%)	23 (20.0%)	99 (22.5%)	0.455
Yes	249 (76.6%)	92 (80.0%)	341 (77.5%)	
Could easily access naloxone if needed				
No	147 (45.2%)	52 (45.2%)	199 (45.2%)	0.998
Yes	178 (54.8%)	63 (54.8%)	241 (54.8%)	
Criminal legal system outcomes	*327 (73.8%)*	*116 (26.2%)*	*N = 443*	
Concerned about getting into trouble with the law if called 911 for an overdose or drug-related health issue				
No	273 (83.7%)	86 (74.1%)	359 (81.2%)	0.023
Yes	53 (16.3%)	30 (25.9%)	83 (18.8%)	
Stopped by law enforcement in past year				
None	127 (38.8%)	21 (18.1%)	148 (33.4%)	< 0.001
At least once	200 (61.2%)	95 (81.9%)	295 (66.6%)	
Searched by law enforcement in past year				
None	240 (73.4%)	46 (39.7%)	286 (64.6%)	< 0.001
At least once	87 (26.6%)	70 (60.3%)	157 (35.4%)	
Sent to jail at least once in past year				
No	252 (77.1%)	50 (43.1%)	302 (68.2%)	< 0.001
Yes	75 (22.9%)	66 (56.9%)	141 (31.8%)	

Table Notes: Numbers and percentages are reported using the analytic sample sizes based on outcome categories in gray. P-values were calculated based on Pearson chi-square tests.

### 3.3 Criminal legal system engagement

Among PWUD on community supervision, 82% reported having been stopped by law enforcement in the past year (Table 2) with a reported median of 4 stops. Nearly three in five PWUD on community supervision reported being searched by law enforcement (60%) and housed in jail at least once (57%) in the past year. Among PWUD on community supervision who were sent to jail, 25% had been sanctioned for drug use.

**Fig. 3:**
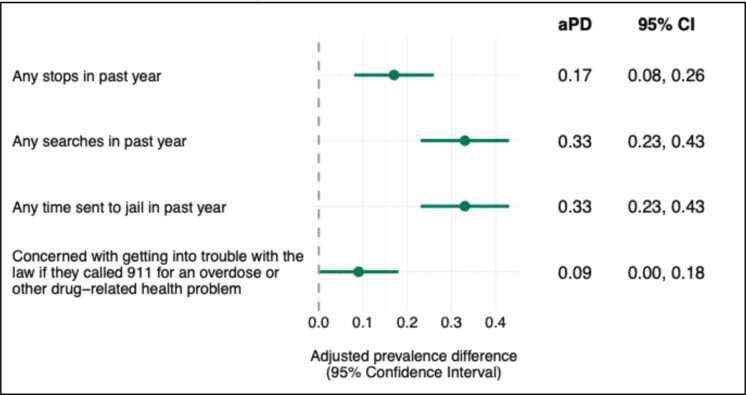
**Average marginal associations between any community supervision and law enforcement interactions in past year (n = 443),** notes: Multivariable identity-binomial models were conducted to estimate prevalence differences. Separate models were run for each outcome and each model adjusted for age, gender, race, and county of interview.

Compared to PWUD who were not on community supervision in the past year, PWUD on supervision had a 17-percentage point higher prevalence of getting stopped or questioned by law enforcement at least once in the past year (Fig. 3, 95% CI: 0.08, 0.26). PWUD on community supervision had a 33-percentage point higher prevalence of getting searched by law enforcement at least once in the past year (95% CI: 0.23, 0.43). Finally, PWUD on community supervision had a 33-percentage point higher prevalence of spending any time in jail at least once in the past year (95% CI: 0.23, 0.43) and had a 9-percentage point higher prevalence of being concerned with getting into trouble with the law if they called 911 for an overdose or other drug-related health issue (95% CI: 0.00, 0.18).

## 4 Discussion

Despite drugs being decriminalized in Oregon during the study period, this sample of PWUD on community supervision were commonly stopped by police (82%), searched (60%), and jailed (57%). Henderson et al. (2024) reported that Oregon’s “annual average search rate per 100 stops from July 2019 to December 2020 was 2.6%, which fell to 1.9% in 2021, 1.6% in 2022, and in the first six months of 2023, it was 1.3%.” Stops, searches, and seizures are used by police to acquire informants to obtain information about criminal networks in exchange for immunity or lower legal infractions (AOUSC, 2016, Hedden-Clayton et al., 2025). The omission of individuals under community supervision within M110’s legislation made this legacy population in Oregon vulnerable to higher CLS engagement, particularly given law enforcement’s practice of running warrants on scene during overdoses or interactions with PWUD (Deonarine et al., 2016, Good et al., 2025, Smiley-McDonald et al., 2022). Qualitative research across four Oregon counties illuminated that some law enforcement targeted people on probation and parole following M110’s enactment to build felony drug cases (Smiley-McDonald et al., 2023).

Our study found that PWUD on community supervision had higher prevalence of past year opioid-related overdose compared to their counterparts. Compared to the general population, individuals on probation have been shown to be 15 times more likely to die from opioid related mortality (361 vs. 23 per 100,000 population) (Boulger et al., 2022). With high rates of overdose or witnessed overdose among community supervision populations (Boulger et al., 2022, Cropsey et al., 2013, Wakeman et al., 2009), it is concerning that 26% of our PWUD sample on community supervision feared they would get in trouble if they called 911 to report a suspected overdose. Oregon’s 2015 GSL explicitly indicates immunity for people who contact emergency medical services for a drug-related overdose and who are on pretrial release, probation, post-prison supervision, or parole (Oregon Laws, 2015, 2015). Our findings comport with other studies showing that despite GSLs, PWUD are reluctant to call 911 for suspected overdoses due to concerns about legal repercussions, including arrest and incarceration in the U.S. and Canada (Baker et al., 2024, Banjo et al., 2014, Byles et al., 2024, Kievit et al., 2022, Latimore and Bergstein, 2017, Moallef et al., 2021, Schneider et al., 2020, van der Meulen et al., 2021, Wettemann et al., 2025), particularly among those who are on probation and parole (Follett et al., 2014, Koester et al., 2017, Wakeman et al., 2009). A recent assessment of GSLs in all 50 states and the District of Columbia concluded that the impact of GSLs has been limited because they do not provide protection from other laws that criminalize “poverty such as trespassing, loitering, vagrancy, and public intoxication” or provide outstanding warrant protections (Lieberman and Davis, 2025). Police exercise substantial discretion on the scene of overdoses, creating vast opportunities for inconsistent practices despite GSLs (Bailey et al., 2025, Del Pozo et al., 2024, Dickson-Gomez et al., 2025, Pamplin et al., 2023, Smiley-McDonald et al., 2022, van der Meulen et al., 2021, Xavier et al., 2022). One in five U.S. law enforcement agencies report that incarceration occurs at least some of the time when there are overdose-related calls for service (Richardson et al., 2023). An Indianapolis-based study revealed one out of every ten overdose survivors was jailed within 6-hours of EMS arrival on the scene, which was nearly four times higher than that of all EMS incidents (Ray et al., 2022). GSLs that protect against arrests are associated with lower rates of overdose in a national county-level study, suggesting that these particular GSLs increase the likelihood people will contact 911 when an overdose occurs (Hamilton et al., 2021). Consequently, some have recommended strengthening GSLs to prevent police running warrants, conducting searches, and arrests on the scene of overdoses (Koester et al., 2017, Smiley-McDonald et al., 2022), while others called for broader reforms for 911 dispatcher laws and practices (Neusteter et al., 2019) or recommended removing police from suspected overdose response altogether (van der Meulen et al., 2021). Our findings strongly support these recommendations recognizing that the fear of calling 911 because of potential and real legal repercussions will persist as long as protections from arrest are absent from GSLs, law enforcement attendance at emergency medical calls for overdose is a possibility, and policing practices continue to be inconsistent due to on scene discretion.

The majority (80%) of community supervised PWUD reported receiving naloxone in the past year and that they could easily obtain it, regardless of community supervision status. This finding may reflect a success of M110 since its enactment provided resources and infrastructure to support harm reduction efforts in all of Oregon’s counties, including naloxone distribution. Over 724,000 naloxone doses have been distributed across Oregon since 2020 (Saves Lives Oregon, 2025). Previous qualitative work completed in 2021 (and prior to M110’s full implementation of its Behavioral Health Resource Networks) showed that naloxone was in short supply as fentanyl flooded Oregon’s unregulated drug markets (Shin et al., 2022). Expanding the number of jails distributing naloxone at release (Tatara et al., 2024, Wenger et al., 2019), and incorporating mail-based overdose education and naloxone distribution (OEND) programs could further reduce overdose mortality among community corrections populations. Mail-based OEND provide an effective way to ensure people who are under community supervision with a mailing address have access to naloxone, particularly those who live in rural areas, and may promote future engagement in treatment (Oser et al., 2025). However, this recommendation may have limited effect in Oregon, which has high rates of people who are unhoused relative to other states. Expanding low-barrier naloxone access through vending machines or naloxboxes could help address the needs of populations with unstable housing (Reid et al., 2023, Russell et al., 2023).

The coda to Oregon’s partial drug decriminalization under M110 is that in response to mounting political pressures, Oregon’s legislature passed House Bill 4002 (HB4002) which reestablished criminal penalties for possession of small amounts of controlled substances as misdemeanors in 2024. Although M110 was never designed to provide people on community supervision access to the lessened harms associated with partial drug decriminalization, it is this very population that is expected to soar in the aftermath of HB4002. Oregon recently forecasted a 46% increase in demand for probation and a 7% increase for parole, and for local supervision to double over the next 5 years due in part to HB4002 (Department of Administrative Services, 2025).

### 4.1 Limitations

Potential methodological limitations of our study should be considered when interpreting our findings. First, the data were self-reported and may be subject to reporting bias. This was a cross-sectional survey, which means causal relationships cannot be established. Our study sample included PWUD in eight Oregon counties and participants were not randomly selected. PWUD are difficult to sample randomly since drug use is heavily stigmatized even in jurisdictions with drug decriminalization policies (Ali et al., 2025). Our study team relied on partner organizations to identify potential participants, which included recruitment at homeless encampments, in public locations where PWUD congregate, and locations where harm reduction supplies and services are provided. Given this sampling strategy, there may be some selection bias because a high proportion of our sample was unhoused or homeless and thus had a higher level of visibility to law enforcement. Finally, we did not ask participants to share what kind of charges were associated with their community supervision status, but it is highly unlikely that they were solely drug possession offenses. We also did not ask participants to describe the conditions of their release, including whether they were mandated to abstain from any drug use. However, 63% of our PWUD sample reported that they would be sanctioned if the CLS found out about their use or possession of drugs, so we are fairly confident that drug use abstinence was likely mandated for most of our sample.

## 5 Conclusion

Despite making up nearly 70% of the U.S. criminal legal system, people on community supervision are often overlooked by policymakers and researchers. The urgency of our findings is clear for Oregon, where probation and parole numbers are projected to increase, but their significance reaches beyond its borders. People under community supervision face more frequent encounters with law enforcement, significantly elevated overdose risks, and a persistent fear of seeking emergency help. These realities highlight the intersection of health vulnerabilities and legal threats confronting community-supervised PWUD and call for immediate action to decrease police presence at overdose scenes following a call for service, enhancing support services to facilitate reentry and recovery, and ensure access to life-saving services like drug treatment and naloxone for this high-risk group.

Finally, if the aim of criminal justice reform is to establish policies that address inequities, reduce harm, and advance public health, lawmakers should ensure that any new drug decriminalization law explicitly include individuals under community supervision under its protections. Failing to do so will perpetuate inequities among those on probation and parole, leaving behind a vulnerable population exposed to heightened and substantial health and legal risks.

## CRediT authorship contribution statement

**Lynn D. Wenger:** Writing – review & editing, Writing – original draft, Supervision, Project administration, Methodology, Investigation. **Esther O. Chung:** Writing – review & editing, Writing – original draft, Visualization, Methodology, Investigation, Formal analysis, Data curation, Conceptualization. **Hope M. Smiley-McDonald:** Writing – review & editing, Writing – original draft, Visualization, Supervision, Methodology, Investigation, Conceptualization. **Barrot H. Lambdin:** Writing – review & editing, Supervision, Project administration, Methodology, Investigation, Funding acquisition, Conceptualization. **Gillian Leichtling:** Writing – review & editing, Writing – original draft, Supervision, Project administration, Methodology, Investigation, Data curation, Conceptualization. **Danielle Good:** Writing – review & editing, Writing – original draft, Supervision, Project administration, Methodology, Investigation, Data curation, Conceptualization. **Alex H. Kral:** Writing – review & editing, Supervision, Project administration, Methodology, Investigation, Funding acquisition, Formal analysis, Data curation, Conceptualization.

## Funding sources

The overall study was supported by 10.13039/100014848Arnold Ventures (Grant 21–06410). The content is solely the responsibility of the authors and does not represent the official views of Arnold Ventures, which had no input into any aspect of the methods, results, or conclusions of this research.

## Disclosure statement

No potential conflict of interest was reported by the authors.

## Declaration of Competing Interest

The authors declare that they have no known competing financial interests or personal relationships that could have appeared to influence the work reported in this paper.
